# Pollinator Dependency and Regional Climate Affect Crop Yield Development Under Climate Change

**DOI:** 10.1002/ece3.73751

**Published:** 2026-06-03

**Authors:** Paula Prucker, Johannes Kollmann, Sara Diana Leonhardt

**Affiliations:** ^1^ Restoration Ecology, TUM School of Life Sciences Technical University of Munich Freising Germany; ^2^ Plant‐Insect Interaction, TUM School of Life Sciences Technical University of Munich Freising Germany

**Keywords:** agricultural production, climate change, ecosystem services, entomophilous crops, insect pollination, pollinator dependency, yield stability

## Abstract

Climate change is severely impacting insects and flowering plants in different ways. While effects on plants, pollinators and their interactions have been amply discussed, subsequent impacts on pollination are rarely assessed. Understanding climate‐change effects on pollination is particularly important for insect‐pollinated crops as it ultimately influences yield and hence food production. This study investigated crop‐yield changes over 35 years in two climatically distinct regions in Germany (cool‐moist vs. warm‐dry). Yield differences among crops showing different levels of pollinator dependency (none, moderate, strong) were analysed in correlation with time (indirect climate‐change measure) and a composite climate parameter (direct measure). Despite rising temperatures and droughts, yields in both regions increased over time across pollinator‐dependency classes, likely due to increased productivity through technological advances. Marked differences in yield optima based on pollinator dependency and region were found in complementary, time‐independent climate correlations. Long‐term average values exceeded optimal yield conditions for moderately pollinator‐dependent plants. Surprisingly, crops that strongly depend on pollinators showed increased yields with warmer, drier conditions, possibly due to fewer late frost events and climate‐driven pollinator community shifts. *Synthesis and applications.* While further research addressing current limitations is needed, the results suggest future crop yields may become less stable despite technological advances, as climatic optima are exceeded for several crops. Additionally, the response of pollinator‐dependent crops to progressing climate change strongly varies depending on the degree of dependence, emphasising the importance of considering these factors in yield predictions and climate adaptation strategies.

## Graphical abstract

This study addresses the question if and how climate change, with rising temperatures and increasing risks of drought events, has affected the yield of entomophilous, that is, insect‐pollinator‐dependent, crops in the past decades. We found weaker yield increases with warming. While climatic yield optima of many crops have been exceeded, strongly pollinator‐dependent crops benefited from warming, likely through adapted pollinators and reduced frost risks.

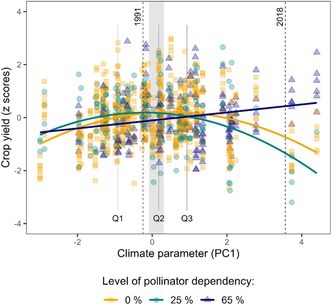

## 1 Introduction

Agricultural crop yields are shaped by both environmental factors and agricultural management. Practical considerations affecting crop yields typically include cultivation methods, such as crop rotation or tillage, and agricultural technologies, such as input application or crop monitoring (Behnke et al. 2018; Yost et al. 2016). The extent to which crops respond to these management strategies is mediated by the plants' plasticity, which determines their capacity to adjust to variable growing conditions and is often driven by cultivar‐specific differences (Nicotra et al. 2010; Zeleke and Nendel 2016). Despite continuous advances in agricultural management and breeding, plant performance remains constrained by environmental factors, particularly temperature, water availability and soil conditions (Lobell et al. 2011; Tölle et al. 2026). As climate change is projected to cause more frequent and intense heat waves and periods of drought (Meehl and Tebaldi 2004; Spinoni et al. 2017), these environmental limits are expected to become even more restrictive. Future climatic conditions will exceed (or are already exceeding) the tolerance range of crops, which can lead to a stress‐imposed reduction in plant fitness (Alqudah et al. 2011). The resulting increase in yield fluctuations or decrease in productivity pose challenges for the agricultural sector, for example, in the form of unstable crop markets (Challinor et al. 2014; Cohen et al. 2021; Lobell and Gourdji 2012; Porter and Semenov 2005; Ray et al. 2015). Beyond these abiotic drivers, biotic interactions play a decisive role in determining yields. In particular, pollinators are crucial for the seed or fruit set of many cultural plants (Klein et al. 2007). While wind‐ or self‐pollinated crops primarily depend on environmental conditions, entomophilous crops additionally rely on effective pollination by flower‐visiting insects to successfully develop high‐quality fruit (e.g., Klatt et al. 2014; Wietzke et al. 2018).

The actual importance of insects as pollen vectors differs among entomophilous crop plants and cultivars (Bishop and Nakagawa 2021; Garratt et al. 2014; Klein et al. 2007; Leonhardt et al. 2013). Field peas, for example, do not benefit from insect pollinators (Klein et al. 2007). Apple fruit set on the other hand is generally greatly enhanced by insect pollination, but the degree of dependence on pollinators regarding fruit quality is variety‐specific (Garratt et al. 2014; Klein et al. 2007). Nevertheless, pollinator exclusion experiments revealed that insect pollination also increased fruit set in crops with only moderate pollinator dependency, such as rapeseed or strawberries (Bartomeus et al. 2014; Gemmill‐Herren and Ochieng' 2008; Klein et al. 2007; Leonhardt et al. 2013; Wietzke et al. 2018). Overall, a significant proportion of crops depend on or benefit from pollination by insects, some with high relevance for human food consumption and nutrient supply, for example, many fruits (Eilers et al. 2011; Klein et al. 2007). In fact, from 1991 to 2009, insect pollination accounted for 12% of the annual economic value of European crop production (Leonhardt et al. 2013). Using a modelling approach, Feuerbacher et al. (2025) further demonstrated that a hypothetical loss of pollinators by 2030 could reduce European crop yields by approximately 8%.

A synthesis of field and mesocosm experiments demonstrated that high pollinator functional diversity and abundance increased crop pollination and plant yield (Woodcock et al. 2019). At the same time, intensive agricultural management and climate change negatively affect insect pollinator populations, abundance and diversity (Hallmann et al. 2017; Ollerton et al. 2014; Raven and Wagner 2021; Vanbergen and The Insect Pollinators Initiative 2013; Vasiliev and Greenwood 2021; Wagner et al. 2021). As ectothermic organisms with only a few options for short‐term heat adaptation, insects are vulnerable to changes in temperature (Paaijmans et al. 2013). Depending on the position and range of the species‐specific climatic niche, they are more or less likely to experience climate‐induced stress, that is, conditions beyond their tolerance levels (Ganuza et al. 2022; Kühsel and Blüthgen 2015). Increased temperatures can therefore change daily and seasonal activity of individual species and affect the abundance, diversity and assemblage of pollinator communities (Forrest 2015; Ganuza et al. 2022; Hegland et al. 2009; Huey and Kingsolver 2019; Kühsel and Blüthgen 2015; Villalpando et al. 2009). Moreover, while direct effects of heavy precipitation on pollinators remain poorly understood, rainfall per se is known to alter pollinator behaviour by reducing overall activity and disrupting flight routes and thus potentially food supply (Lawson and Rands 2019). Furthermore, existing metabolic adaptations to seasonally cold conditions are challenged in warming winters with repeated cycles of freezing and thawing weather (Bale and Hayward 2010), further challenging insect populations.

Climate change, and here predominantly drought, can affect flower morphology and reduce numbers and sizes of flowers, colour signal strength, or the quantity and quality of pollen and nectar (Carroll et al. 2001; Descamps et al. 2021; Phillips et al. 2018; Ziska et al. 2016). As a consequence, stressed plants may be less attractive to insects and thus less likely to be pollinated (Alqudah et al. 2011; Carroll et al. 2001; Descamps et al. 2021; Gallagher and Campbell 2017; Phillips et al. 2018). The subsequent reduction in plant fitness may be further exacerbated through decreased pollinator availability in agricultural landscapes. Moreover, plants and pollinators can differ in the level of adaptation to climate shifts which results in phenological mismatches (Forrest 2015; Kehrberger and Holzschuh 2019; Polce et al. 2014).

Increasing temperatures and droughts thus pose challenges for crop pollination and ultimately food production. However, research on climate‐change impacts in agricultural plant production has so far mainly focused on overall economic implications (e.g., monetary losses resulting from yield variability), single crops (e.g., winter wheat) or specific management systems, such as fruit tree orchards (Bönecke et al. 2020; Lobell and Field 2007; Polce et al. 2014; Ray et al. 2015). It is therefore poorly understood whether climate differently affects the yield of (non‐)entomophilous crop plants. Given that studies on long‐term trends in crop production predicted an increase in pollinator dependency of crops worldwide (Aizen et al. 2008, 2009), while insects, including pollinator populations, are declining (Raven and Wagner 2021; Vasiliev and Greenwood 2021), it is likely that pollinator‐dependent crops suffer twofold from climate change, because their yield is threatened by both direct abiotic effects of climate change on the plants themselves and indirect effects through weakening their mutualistic relationship with pollinators (Trunschke et al. 2024).

Understanding how climate change and pollinator dependency interactively affect crop yields is essential for the development of responsive strategies to ensure food security. To gain insight into the past and current trends of crop yield under climate change, and to assess whether entomophilous and non‐entomophilous crops respond differently, yield data from 1985 to 2019 for major crops in two climatically contrasting German regions, that is, cool‐moist vs. warm‐dry, were analysed and compared. The regions were selected in a space‐for‐time approach, as the warm‐dry region may represent climatic conditions as assumed for the cool‐moist region in the future. An overall decrease in yield over the years was expected due to accelerating climate change. A stronger reduction in the yield of entomophilous crops, compared to non‐entomophilous crops, was further predicted because of pollination limitation mediated by climate‐induced changes in pollinator communities. Overall, stronger trends were anticipated for the warm‐dry region compared to the cool‐moist region.

## 2 Materials and Methods

### 2.1 Study Region

The federal state of Bavaria is located in the south‐east of Germany, with an elevation gradient from warm‐dry Lower Franconia (LF) in the north to cool‐moist Upper Bavaria (UB) in the south (Bavarian Surveying and Mapping Authority [BVV] and German Federal Agency for Cartography and Geodesy [BKG] 2025). At their midpoints, the two regions are approximately 240 km apart, while the closest distance between two administrative borders is 85 km (BVV and BKG 2025). Landscape composition and configuration are similar, with 34% and 40% forested areas and 45% and 43% agricultural areas in UB and LF, respectively (Bavarian State Ministry for Food‚ Agriculture and Forestry [StMELF] 2022). Typical crops cultivated in both regions comprise cereals (e.g., wheat, rye, barley; representing 35% of the total area set aside for agricultural and fruit crops in 2023), maize (14%), oil seeds (e.g., rapeseed, sunflowers; 8%), root crops (e.g., potatoes, turnips; 3%), legumes (e.g., field peas, broad beans; 1%), and fruit crops (e.g., apples, pears, plums, cherries; < 1%) (Bavarian State Statistical Office [LfStat] 2024a, 2024b).

The mean annual temperature was 8.2°C in UB, and 8.5°C in LF between 1971 and 2000 (Bavarian State Ministry of the Environment and Consumer Protection [StMUV] 2021). During the same period, LF experienced 59% more heat days (*T*_max_ ≥ 30°C) and 12% more summer days (*T*_max_ ≥ 25°C) compared to UB; conversely, UB had 10% more frost days (*T*_min_ ≤ 0) and 21% more ice days (*T*_max_ ≤ 0) than LF (StMUV 2021). Climate models predict a state average temperature increase of 1.1°C until the year 2100 in the best case (i.e., ambitious climate mitigation efforts aligned with the ‘Paris Agreement’), and 3.8°C in the worst case (unabated climate change), along with an 11%–13% decrease in summer rain for the 2nd scenario with no action taken against climate change (StMUV 2021).

Long‐term data on pollinator communities are, to our knowledge, not available in the spatial and temporal resolutions necessary to infer and compare trends between the two focal regions. However, for 2019, Malaise trap data published by Uhler (2021) found a generally lower total insect biomass in UB than in warm‐dry LF, whereas overall richness was similar across the two regions (Figure S1A,B). For bees, butterflies and flies, three highly important pollinator groups known to pollinate crops typically grown in both regions, that is, rapeseed and apple, for example (LfStat 2024a, 2024b; Klein et al. 2007), region effects varied. Overall Hymenoptera richness was similar in the two regions, while a higher diversity of Diptera was found in cool‐moist UB and more different Lepidoptera records were made in warm‐dry LF (Figure S1C,D,E).

### 2.2 Data Processing

#### 2.2.1 Yield Data and Pollinator Dependency

Data on field crop yield, for example, wheat, corn, barley etc., were obtained from agricultural economics monitoring of the Bavarian State Institute for Agriculture for the years 1985–2019 (R. Schätzl, J. Reisenweber and M. Schägger, personal communication, 29 July 2021). Data on fruit yield, for example, apples, cherries, plums, etc. (from here on also called ‘crop’), were obtained from the German federal and state statistical offices' database for the period 2003–2019 (LfStat and Bavarian State Office for Statistics and Data Processing [LfStaD] 2021). The data was separately processed for each region and represented as the sum of yield in decitons per hectare (dt ha^−1^) recorded for a specific year across the whole region. Only crop types with economic interest in their fruits resulting from pollination were included (Table 1); therefore, green fodder as well as root crops, for example, potatoes and turnips, were excluded. Crops with data entries for only a few years, that is, berry crops including strawberries, currants and raspberries, as well as data on mix crops, for example, mixed cereals, were not considered.

**TABLE 1:** Analysed crop types with pollinator dependency and cultivation area relative to the total agriculturally used area in Bavaria.

Crop type	Pollinator dependency	Area (%)
Cereal crops		35
Durum wheat	None (0%)	
Maize	None (0%)	
Oats	None (0%)	
Rye	None (0%)	
Spring barley	None (0%)	
Spring wheat	None (0%)	
Triticale	None (0%)	
Winter barley	None (0%)	
Winter wheat	None (0%)	
Maize	None (0%)	14
Oil seeds		8
Summer rapeseed	Moderate (25%)	
Sunflower	Moderate (25%)	
Winter rapeseed	Moderate (25%)	
Legumes		1
Broad beans	Moderate (25%)	
Field peas	None (0%)	
Fruit crops		< 1
Apples	Strong (65%)	
Mirabelles	Strong (65%)	
Pears	Strong (65%)	
Plums	Strong (65%)	
Sour cherries	Strong (65%)	
Sweet cherries	Strong (65%)	

*Note:* Pollinator dependency levels were assigned following Klein et al. (2007) and Leonhardt et al. (2013). Data on area proportions were calculated for the exemplary year 2023 and taken from the Bavarian State Statistical Office [LfStat] (2024a, 2024b).

To achieve comparability among the different crop yield magnitudes, yield and fruit data were *z*‐standardised for each crop type and region across years (i.e., rescaled to a mean of 0 and a standard deviation of 1). Following Klein et al. (2007) and Leonhardt et al. (2013), different crops were characterised by their insect pollinator‐dependency levels as opposed to the other regionally relevant wind and self‐pollination mechanisms, using the three categories ‘no pollinator dependency’ (0%, e.g., durum/spring/winter wheat, field peas, maize, oats, rye, spring/winter barley, triticale), ‘modest pollinator dependency’ (25%, e.g., broad beans, sunflower, summer/winter rapeseed) and ‘strong pollinator dependency’ (65%, e.g., apples, mirabelles, pears, plums, sour/sweet cherries; Table 1).

#### 2.2.2 Climate Data

Climate data was retrieved from the Open Data Server of the German Meteorological Service (German Meteorological Service [DWD] Climate Data Center 2021) for all available stations within the two regions, focusing on the main crop production areas of each region and thus excluding uplands and alpine areas. Records included data on daily average temperature *T*_avg_ [°C], maximum temperature *T*_max_ [°C], minimum temperature *T*_min_ [°C] and amount of precipitation P [mm]. The dataset was trimmed to entries between 1985 and 2019 to match the range of yield data entries and included only records for the vegetation period of the crops included (i.e., March–October). Dry days D (P < 1 mm) were identified based on the data entries on precipitation. In case of incomplete recording over a month, the records for dry days were extrapolated by dividing the sum of dry days for a specific month by the total number of records made and multiplying this ratio by the total number of calendar days for this specific month. Mean values for each year and region were calculated by summarising the number of dry days and averaging the values for *T*_avg_, *T*_max_, *T*_min_ and P per month of a year and station in a region.

### 2.3 Statistical Analysis

Statistical analyses were conducted in RStudio (version 1.3.1093, released by RStudio PBC) using the R language (R Core Team 2023). To obtain a single composite climate parameter that can be related to climate change, a Principal Component Analysis (PCA; Figure S2) was performed on the climate data using the ‘prcomp’ command of the R package ‘stats’ (R Core Team 2023) and retrieved PC1 and PC2 as variables summarising overall climatic effects. Both axes displayed significant correlations with all original climate parameters (Figure S3 for PC1; R package ‘correlation’, Pearson method; Makowski et al. 2020). PC1 accounted for 54% of the variance in the data and was strongly positively correlated with *T*_avg_, *T*_max_, *T*_min_ and D, and negatively with P (Figure S3). PC1 can thus be used as a time‐independent parameter describing the progression of climate change leading to increased temperatures and drought following a conservative modelling approach with assumed linear trends. PC2 explained 34% of the variance in the data and most strongly correlated with *T*_min_ and P (Figure S2).

Linear models (command ‘lm’ of R package ‘stats’; R Core Team 2023) were fitted to assess the overall effects of (1) time (year) and (2) climate change (PC1), as well as their interactive effects with region and pollinator dependency on crop yield. In the year‐based model, the interpretation of the main effect of dependency is constrained by the data structure, as one dependency class covered only a limited portion of the overall study period. In the models testing for time effects, time was included as a simple (only linear) explanatory variable. The final models testing for climatic conditions additionally included PC1 as a quadratic explanatory variable and were simplified to only include a linear PC1 variable in case no significant optimum relationship was detected. Crop yield trajectories of relevant subgroups were obtained by fitting separate linear models on data subsets and compared in a pairwise manner using ‘emtrends’ for linear trends (year) and ‘emmeans’ on quartile means for quadratic trends (PC1). The same modelling approach was used on crop‐level data subsets to graphically compare crop‐specific yield trajectories with the observed global trends and to assess the contribution of single crop types to the overall patterns (Supporting Information 2).

To additionally explore whether the occurrence of years of below‐ or above‐average yields varied between pollinator dependency groups and climatic conditions, the distribution of years with high‐ and low‐yields for the dataset's climatic range was assessed. For this, yearly mean yield values for each region and dependency group were compared to the regional mean yield value across years and dependency groups. Data points were further grouped into PC1 values above, within, or below the regional average (defined as the range around the regional PC1 mean value representing 1/3 of the overall regional PC1 range) to assess the relative proportion of average and extreme years within the yield and climatic range.

## 3 Results

### 3.1 Crop Yields Changed Over Time in Both Regions

**FIGURE 1:**
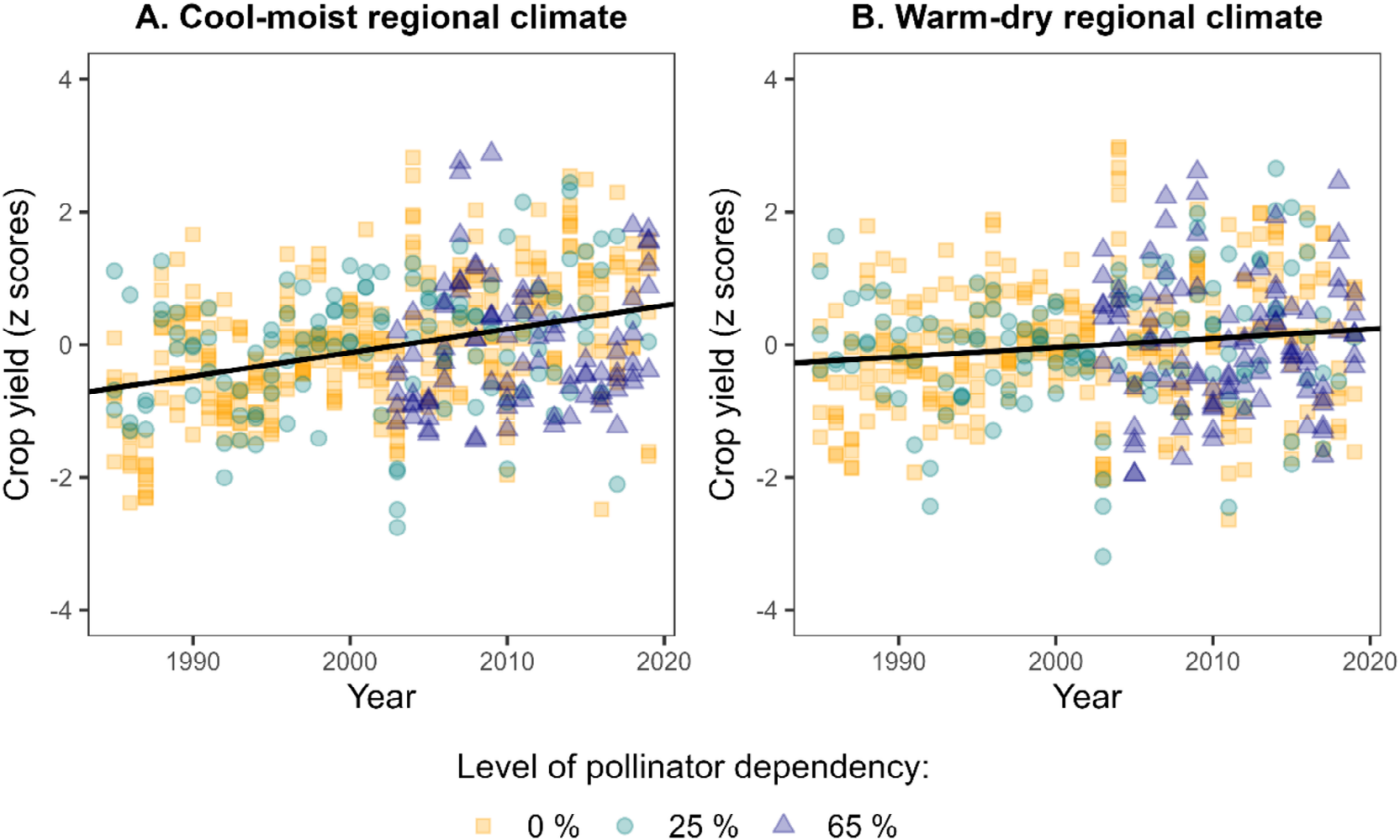
General trends in crop yield over time of moderately (green points) and strongly (blue triangles) pollinator‐dependent crops, as well as pollinator‐independent crops (orange squares) in two climatically different regions in Germany. Yield increase over time is less pronounced in warm‐dry regional climate (*R* ^2^ = 0.02, *n* = 514) compared to cool‐moist regional climate (*R* ^2^ = 0.12, *n* = 533). Linear modelling using ‘lm’ from the R ‘stats’ package (R Core Team 2023). Data sourced from LfStat and LfStaD (2021) and R. Schätzl, J. Reisenweber and M. Schägger (personal communication, 29 July 2021).

Crop yields in both regions changed over time, and the effect differed between regions (Figure 1, Table 2). In both regions, crop yields generally increased over time, irrespective of crop pollinator dependency. However, yield increases were significantly weaker in the warm‐dry region than in the cool‐moist region (*p* = 0.042), with annual increases of +1.4% ± 10.2% in the warm‐dry region and +3.6% ± 9.7% in the cool‐moist region. The observed overall trends were mainly driven by maize, rye, and rapeseed, while fruit trees, field peas, and broad beans displayed unclear or negative trends (Supporting Information 2, Figure S4).

**TABLE 2:** Summary of single and interactive statistical effects of year (i.e., time), pollinator dependency and region on crop yields (*R* ^2^ = 0.08, *n* = 1047).

Effect	*F*	df	*p*
Year	66.1	1	**< 0.001**
Pollinator dependency	5.4	2	**< 0.01**
Region	0.1	1	0.83
Year * Pollinator dependency	2.0	2	0.14
Year * Region	11.4	1	**< 0.001**
Dependency * Region	1.5	2	0.22
Year * Pollinator dependency * Region	0.6	2	0.55

*Note:* Linear modelling using ‘lm’ and ‘anova’ from the ‘stats’ package (R Core Team 2023). Bold values are significant *p*‐values and thus represent significance values themselves.

### 3.2 Optimal Yield Conditions Differed Between Pollinator Dependency and Region

**FIGURE 2:**
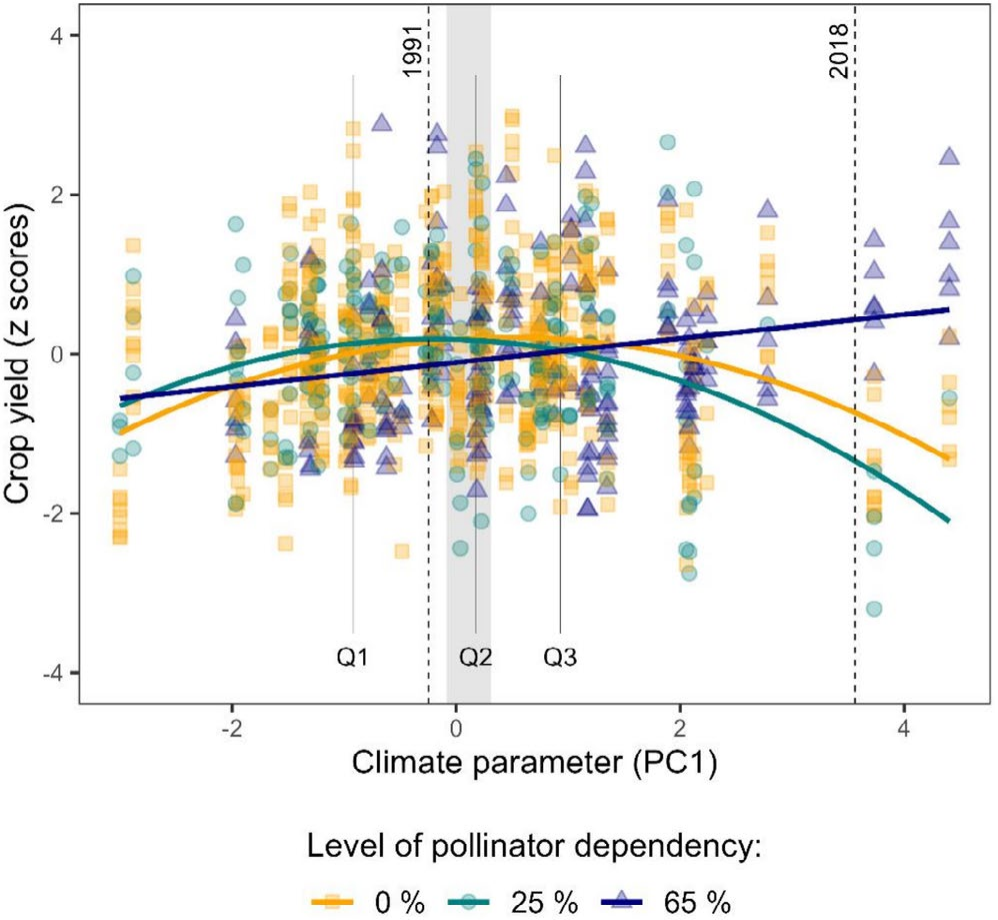
Relationship between climatic conditions and crop yields over time for moderately (green points) and strongly (blue triangles) pollinator‐dependent crops as well as pollinator‐independent crops (orange squares) across two climatically different regions in Germany. Yields of strongly pollinator‐dependent crops are linearly increasing with higher values of PC1 (*R* ^2^ = 0.05, *n* = 194), while long‐term average values exceed optimal climatic conditions for moderately pollinator‐dependent crops (*R* ^2^ = 0.09, *n* = 248), but not for pollinator‐independent crops (*R* ^2^ = 0.08, *n* = 607). Dashed vertical lines indicate regional PC1 average values for 1991 and 2018, 2 years with below and above long‐term average temperature records on national scale, respectively (DWD Climate Data Center 2021). Solid vertical lines in greyscale indicate quartile boundaries, and the grey area indicates regional PC1 average values across the dataset (R Core Team 2023). Linear modelling using ‘lm’ from the R ‘stats’ package (R Core Team 2023). Yield data sourced from LfStat and LfStaD (2021) and R. Schätzl, J. Reisenweber and M. Schägger (personal communication, 29 July 2021). Climate data underlying PC1 values sourced from DWD Climate Data Center (2021).

Climate change (PC1) was significantly correlated with crop yield, revealing trends that varied with pollinator dependency, but not across regions (Figure 2, Table 3). Pollinator‐independent crops showed a clear climatic optimum for yield shifted to the right and thus towards warmer and drier conditions in relation to the long‐term regional average (grey area in Figure 2; curve slope −0.10 ± 0.33, *p* < 0.001). Moderately pollinator‐dependent crops also showed a climatic optimum (curve slope −0.11 ± 0.37, *p* < 0.001), even though optimal yields were shifted to the left and thus slightly cool‐moister conditions, compared to pollinator‐independent crops. Crop yields of strongly pollinator‐dependent crops, in turn, were positively correlated with climate change (PC1), with the highest values at warm‐dry climate (slope +0.03 ± 0.99, *p* = 0.002). These trends resulted in an increasing separation of crop yields among pollinator‐dependency groups towards the upper end of the PC1 gradient. Post hoc comparisons indicated no significant differences among pollinator‐dependency groups in the first and second PC1 quartiles. In contrast, in the third quartile, yields of strongly pollinator‐dependent crops were significantly lower than those of non‐dependent crops (*p* = 0.020). In the fourth quartile, yields of strongly pollinator‐dependent crops were significantly higher than those of moderately pollinator‐dependent crops (*p* = 0.046). Overall, the observed climatic curves closely matched those of wheat and rye and summer rapeseed, while sour cherries, sunflowers and single other cereal crops displayed unclear or slightly shifted trends (Supporting Information 2, Figure S5).

**TABLE 3:** Summary of single and interactive statistical effects of PC1 (i.e., climate change), pollinator dependency and region on crop yields (*R* ^2^ = 0.08, *n* = 1047).

Effect	*F*	df	*p*
PC1^2^	37.0	1	**< 0.001**
Pollinator dependency	0.1	2	0.97
Region	2.7	1	0.10
PC1^2^ * Pollinator dependency	11.1	2	**< 0.001**
PC1^2^ * Region	1.8	1	0.18
Dependency * Region	1.6	2	0.19
PC1^2^ * Pollinator dependency * Region	1.9	2	0.16

*Note:* Linear modelling using ‘lm’ and ‘anova’ from the ‘stats’ package (R Core Team 2023). Bold values are significant *p*‐values and thus represent significance values themselves.

### 3.3 Differences Between Region and Dependency Group Persist for Above‐Average Years

**FIGURE 3:**
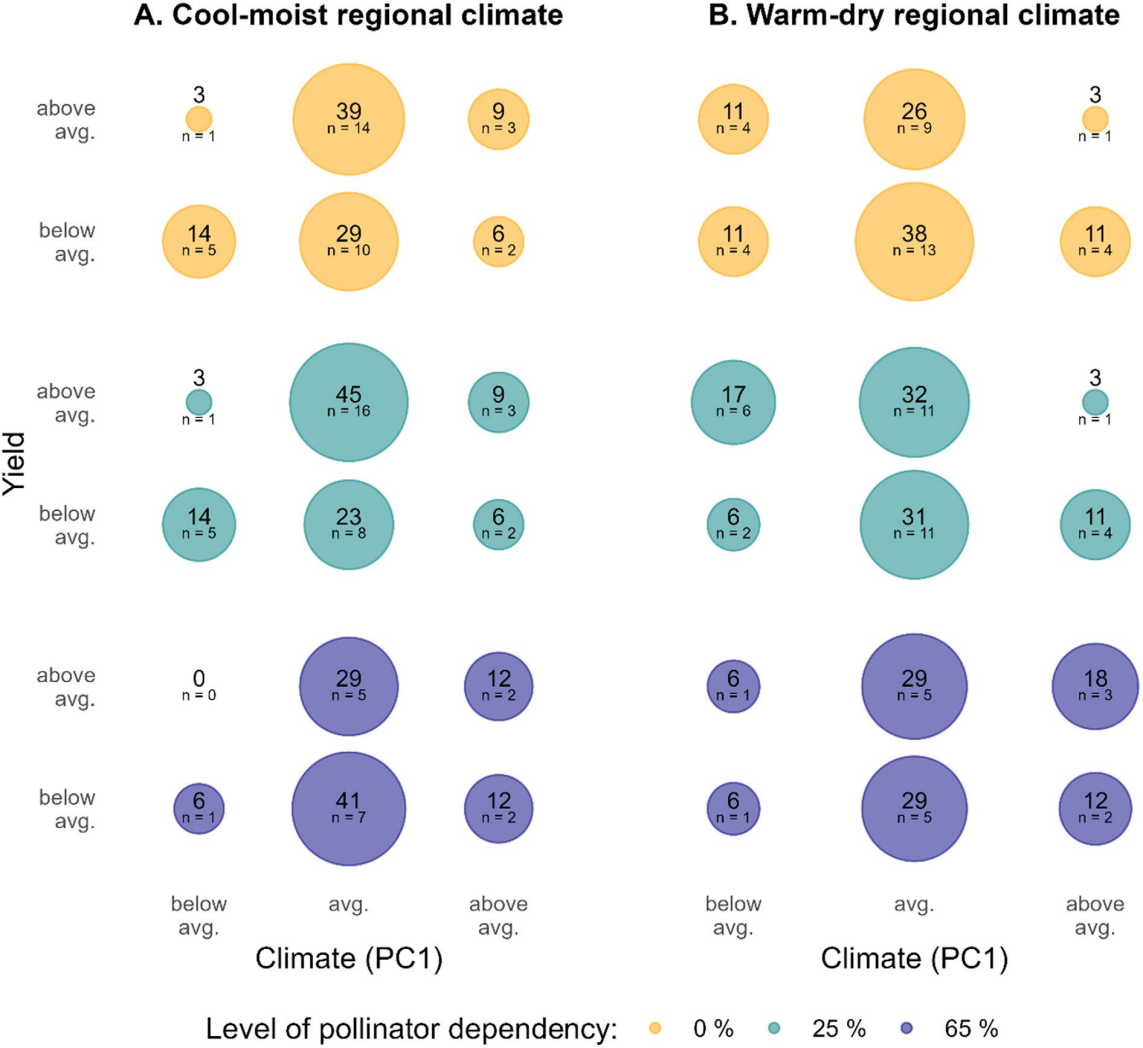
Proportion of years with above and below average yields in relation to below, average and above average climate conditions (PC1) for the two regions. The analysis was done for moderately (green) and strongly (blue) pollinator‐dependent crops, and pollinator‐independent crops (orange). Numbers in circles represent the proportion [%] of years falling in the respective yield and climate category (with the number of years below) and are visualised by circle size. Yield data sourced from LfStat and LfStaD (2021) and R. Schätzl, J. Reisenweber and M. Schägger (personal communication, 29 July 2021). Climate data underlying PC1 values sourced from DWD Climate Data Center (2021).

In the cool‐moist region, years with warm‐drier conditions than on average led to higher yields across crop categories as compared to years with cool‐moister conditions (Figure 3A). In the warm‐dry region, years with warm‐drier conditions than on average had lower yields as compared to years with cool‐moister conditions, but only for moderately dependent and pollinator‐independent crops (Figure 3B). For highly pollinator‐dependent crops, warm‐drier years increased the likeliness of above‐average yields.

## 4 Discussion

### 4.1 Yield Increase Is Affected by Climate Change

Decreasing yields of pollinator‐dependent crops over the past decades were predicted due to simultaneously recorded pollinator declines, potentially resulting in pollination limitation. In contrast to expectations, the results showed an increase in crop yields irrespective of pollinator dependency groups over time. This aligns with some published studies and may be attributed to technological advancements and practical improvements in the agricultural sector since the mid 20th century, leading to an increase in productivity per area of cultivated land during the examined period through enhanced machinery, precision agriculture and improved soil and pest management (Lobell and Field 2007; Lobell and Gourdji 2012; Lobell et al. 2011; Rabbinge and van Diepen 2000; Ray et al. 2012). Moreover, traditional breeding methods were complemented by biotechnological innovations in the past decades, such as genomic‐assisted breeding, which has rendered high‐yielding and disease‐resistant crop varieties available for cultivation (Rabbinge and van Diepen 2000; R. K. Singh et al. 2020). This pattern observed for southern Germany is consistent with global studies on crop yield developments and indicates that short‐term gains in productivity may mask longer‐term challenges posed by climate change or the loss of mutualists (Lobell and Field 2007; Lobell and Gourdji 2012; Lobell et al. 2011).

However, comparing yield trends between the climatically contrasting regions revealed a more nuanced picture. The increase in crop yield over time was less pronounced for crops in the warm‐dry region, in particular. Since the two study regions are similar in terms of landscape composition and agricultural management, it is unlikely that the observed differences in yield increase between the two regions were driven by land‐use differences, suggesting in turn that innovation and technology‐driven increases in crop yield may slow down, plateau, or even decrease with climate change. Interestingly, this trend was independent of crop pollinator dependency. This suggests that breeding and optimisation efforts for new crop varieties that show enhanced yield and/or more resilience towards stressors have progressed in a similar manner across crop pollinator‐dependency classes. Breeding advances may even be considered equally effective across crop groups, not only for widely cultivated high‐revenue field crops but also for more demanding niche crops, including broad beans and field peas (see Table 1), and for perennial systems such as fruit trees. For example, pollinator‐dependent fruit trees can benefit from grafting techniques traditionally allowing for a fast introduction of new desirable traits (Mudge et al. 2009), an approach that is increasingly discussed as a means to enhance climate‐change adaptation in vegetable production as well (H. Singh et al. 2020).

### 4.2 Optimal Climate Conditions Are Partly Already Exceeded

Complementing the results from the time‐based analysis, time‐independent correlations between yield and climatic conditions revealed optimum curves for most crops. Moreover, the shape and position of the curves varied with pollinator dependency. For pollinator‐independent and moderately pollinator‐dependent crops, yield optima were found for climate conditions slightly above or below the long‐term average. Hence, their optimal climatic conditions have already been passed with current climate change or will most likely soon reach the tipping point. These clear optimum trends are most likely due to pollination limitations, potentially caused by specific pollinators suffering from climatic stress beyond certain conditions. For example, Zoller et al. (2023) observed climate‐change‐induced reductions in abundance in the subarctic region primarily for hoverflies, which alters entire pollinator communities and subsequently plant‐pollinator interaction networks. Similarly, in a study in tropical and subtropical America, Diptera displayed generally lower climatic tolerance at foraging than hymenopterans (López‐Uribe et al. 2024). Likewise, Kühsel and Blüthgen (2015) found that hoverflies and other flies were most active at lower temperatures as compared to bees (including bumblebees, which are generally considered a cold‐adapted taxon) or butterflies in a temperate region, which may ultimately affect yields of Diptera‐dependent crops due to changes in their communities (Kühsel and Blüthgen 2015; Villalpando et al. 2009). These observations are consistent with those of Uhler (2021; see Supporting Information 1), who reported similar insect biomass across the study regions but higher Diptera richness in the cool‐moist region than in the warm‐dry region. While richness does not directly measure activity, temperature‐driven differences in species richness can mirror underlying activity patterns of taxonomic groups. Even though research on climate‐change‐driven pollination limitation in crops is still scarce, these alterations may affect especially those crops that are at least partly pollinated by insects susceptible to climate change, for example, hoverfly pollination in oilseed rape (Jauker and Wolters 2008).

In contrast, strongly pollinator‐dependent crops showed a distinct positive correlation of yields with progressing climate change. In addition to the significant role of broad beans in shaping the moderately pollinator‐dependent climatic curve ‐ possibly due to their efficient pollination by cold‐adapted bumblebees (Marzinzig et al. 2018) ‐ these distinct patterns among pollinator‐dependent crop yields are likely influenced by the life‐form traits of most crops included in the analysis.

While moderately pollinator‐dependent crops include mainly annual field crops, strongly pollinator‐dependent crops are comprised of pome or stone fruit trees. Fruit trees flower early in the season and are thus susceptible to spring frosts, which can severely damage their flowers and thus reduce the overall fruit set and thus yield (Rodrigo 2000). While climate change generally increases the risk of spring frost by advancing the vegetation period, more recent studies on the effect of climate change on spring frost identified the regional elevation level as a major factor for spring frost risk (Lamichhane 2021; Unterberger et al. 2018; Vitasse et al. 2018).

Fruit trees are typically known for alternating between years of high yields and periods of low fruit production (Herrera et al. 1998), a pattern known as ‘masting’ which is significantly influenced by large‐scale climatic phenomena influencing temperature and precipitation, such as the North Atlantic oscillation (Ascoli et al. 2017). Fruit tree masting events may thus be affected by climate change. Moreover, the significant positive fruit yield development points to an advantage of these crops at sites with a warmer climate, where the risk of spring frost is reduced. Fruit trees are likely also more resilient towards short‐term drought events as compared to annual field crops because they have deep roots which access low water levels.

Fruit trees may further benefit from pollination by insects tolerating or even preferring warmer climatic conditions. For example, bees are globally most abundant and diverse in warm‐dry climate regions (Michener 1979), and are known to be efficient pollinators of fruit trees (e.g., Garratt et al. 2016). Their early flowering time and large resource display may further have led to a concentration of available pollinators on fruit trees, as orchards as mass‐flowering crops represent valuable foraging habitats in spring when meadows, pastures, and verge habitats do not yet provide comparable amounts of resources (Jachuła et al. 2022; Proesmans et al. 2019). If they have access to sufficient water, fruit trees may thus generally benefit from (or be unaffected by) the pollinator community turnover where incoming warm‐adapted taxa outweigh the loss of heat‐intolerant species.

### 4.3 Need to Consider Pollinator Dependency and Regional Climate in Adaptation Strategies

The observed trends suggest that regional climate affected crop yield in the past. They further indicate that, within the study period, fruit set was achieved across crop categories, implying that pollination services were still sufficiently maintained. However, there might be a trend reversal for yield development with yields plateauing or even declining in the future. This may occur earlier for pollinator‐dependent crops and in warm regions than for pollinator‐independent crops and in cool regions. In fact, the results hint towards a tipping point for field crops, in particular moderately pollinator‐dependent crops, likely because climatic optima of both crops and their main pollinators are about to be exceeded. On the other hand, fruit trees, for example, could potentially benefit from progressing climate change, as shifting conditions may become increasingly favourable for this group of crops.

Notably, the presented analysis focused primarily on climate change as a driver of pollinator decline and thus pollination limitation in pollinator‐dependent crops. However, it did not account for individual or additive effects of other stressors such as land use and thus habitat change, agrochemical use and invasive species, which are known to negatively affect pollinator populations and may further compromise yields of pollinator‐dependent crops in the future (Knauer et al. 2026; Nicholson et al. 2024; Potts et al. 2010). In addition, these stressors are often closely linked and can have interactive effects, as in the example of climate change and intensive land use (Ganuza et al. 2022; Raven and Wagner 2021). Moreover, it remains unclear whether the observed yield trends in response to climate change are primarily driven by changes in pollinator activity, plant fitness or a combination of both (Hegland et al. 2009). To disentangle these effects, full‐factorial field‐level experiments are needed to further verify the findings of the presented study, ideally complemented by correlative analyses using data of higher resolution to account for site‐specific covariates. This would allow for the inclusion of locally relevant factors, such as cultivar type incl. reproductive traits and water requirements, land management incl. pest control measures and pollinator support, (a)biotic site conditions, or extreme events resulting from non‐linear climate change developments (Lobell et al. 2009), which could not be considered in the presented analysis.

Despite these limitations, this study underscores the importance of pollinator dependency and regional climate to be included in yield predictions and climate adaptation, by, for example, considering pollinator dependency levels of crops and the climatic tolerances of their main pollinators in yield predicting models. This is essential for climatically vulnerable regions to forecast future pollinator communities and thus pollination services. Given the high variability in and low predictability of yearly climatic conditions and the occurrence of weather extremes and thus crop yields (Porter and Semenov 2005), the importance of maintaining biodiversity, specifically through providing habitats for diverse pollinator communities, needs to be stressed. Only diverse pollinator communities maintain a broad climatic community niche (Kühsel and Blüthgen 2015) and thus ensure that the loss of some pollinators is buffered. Therefore, supporting resilient insect pollinator communities of high species abundance and diversity is key to safeguarding pollination services for crops (Bartomeus et al. 2013; Elmqvist et al. 2003; Senapathi et al. 2015; Steffan‐Dewenter et al. 2005), also under climate change, which poses a significant threat to this important ecosystem service and hence crop yield and ultimately food production.

## Author Contributions

**Paula Prucker:** conceptualization (equal), data curation (lead), formal analysis (lead), investigation (lead), writing – original draft (lead), writing – review and editing (supporting). **Johannes Kollmann:** conceptualization (equal), funding acquisition (equal), writing – review and editing (supporting). **Sara Diana Leonhardt:** conceptualization (equal), funding acquisition (equal), writing – review and editing (lead).

## Funding

This work was supported by Bayerisches Staatsministerium für Umwelt und Verbraucherschutz (TEW01002P‐77745).

## Conflicts of Interest

The authors declare no conflicts of interest.

## Supporting information


**Figure S1:** Malaise trap data collected by Uhler (2021) in 2019 shows (A) similar total insect richness and (B) Hymenoptera richness in the two study regions, that is, cool‐moist Upper Bavaria (UB) and warm‐dry Lower Franconia (LF), while (C) total insect biomass and (D) Lepidoptera richness was higher in LF, and (E) Diptera richness was higher in UB (all *p* < 0.01; *n* = 696–968). Linear modelling using ‘lm’ and ‘anova’ from R package ‘stats’ (R Core Team 2023).
**Figure S2:** Clustering of climate records from the two study regions (cool‐moist UB: light grey, warm‐dry LF: dark grey) with the contribution of climate variables (D = sum of dry days; P = mean precipitation; *T*_avg_ = mean average temperature; *T*_max_ = mean maximum temperature; *T*_min_ = mean minimum temperature) based on their principal components 1 and 2 (A; *n* = 631) cumulatively explaining 88% of variance (B). Principal component analysis using ‘prcomp’ from R package ‘stats’ (R Core Team 2023). Data sourced from DWD Climate Data Center (2021).
**Figure S3:** Strong positive correlation between PC1 values and (A) average temperature, (B) maximal temperature, (C) sum of dry days and (D) strong negative correlation between PC1 and mean precipitation (*n* = 631). Pearson correlation using ‘correlation’ from R package ‘correlation’ (Makowski et al. 2020). Data sourced from DWD Climate Data Center (2021).
**Figure S4:** Trends in crop yield over time of moderately (green points) and strongly (blue triangles) pollinator‐dependent crops, as well as pollinator‐independent crops (orange squares) in two climatically different regions in Germany. Thin lines show the overall trend line for each pollinator dependency group compared to bold lines showing the respective crop type's trend. Solid lines represent significant trends as opposed to dashed lines for insignificant trends. Linear modelling using ‘lm’ from the R ‘stats’ package (R Core Team 2023). Data sourced from LfStat and LfStaD (2021) and R. Schätzl, J. Reisenweber and M. Schägger (personal communication, 29 July 2021).
**Figure S5:** Relationship between climatic conditions and crop yields over time for moderately (green points) and strongly (blue triangles) pollinator‐dependent crops as well as pollinator‐independent crops (orange squares) across two climatically different regions in Germany. Dashed lines indicate regional PC1 average values for 1991 and 2018, 2 years with below and above long‐term average temperature records on national scale, respectively (DWD Climate Data Center 2021), while grey areas indicate regional PC1 average values across the dataset (R Core Team 2023). Thin lines show the overall trend line for each pollinator dependency group compared to bold lines showing the respective crop type's trend. Solid lines represent significant trends as opposed to dashed lines for insignificant trends. Linear modelling using ‘lm’ from the R ‘stats’ package (R Core Team 2023). Yield data sourced from LfStat and LfStaD (2021) and R. Schätzl, J. Reisenweber and M. Schägger (personal communication, 29 July 2021). Climate data underlying PC1 values sourced from DWD Climate Data Center (2021).

## Data Availability

Data are available in the Zenodo repository at DOI: 10.5281/zenodo.20488475.
