# Supplementary material for: Pollinator Dependency and Regional Climate Affect Crop Yield Development Under Climate Change
Source: Ecol Evol. 2026 Jun 3;16(6):e73751. doi: 10.1002/ece3.73751 (PMC13239248; doi:10.1002/ece3.73751)
Supplement: Supplementary file 1 — Figure S1: Malaise trap data collected by Uhler (2021) in 2019 shows (A) similar total insect richness and (B) Hymenoptera richness in the two study regions, that is, cool‐moist Upper Bavaria (UB) and warm‐dry Lower Franconia (LF), while (C) total insect biomass and (D) Lepidoptera richness was higher in LF, and (E) Diptera richness was higher in UB (all p < 0.01; n = 696–968). Linear modelling using ‘lm’ and ‘anova’ from R package ‘stats’ (R Core Team 2023). Figure S2: Clustering of climate records from the two study regions (cool‐moist UB: light grey, warm‐dry LF: dark grey) with the contribution of climate variables (D = sum of dry days; P = mean precipitation; T avg = mean average temperature; T max = mean maximum temperature; T min = mean minimum temperature) based on their principal components 1 and 2 (A; n = 631) cumulatively explaining 88% of variance (B). Principal component analysis using ‘prcomp’ from R package ‘stats’ (R Core Team 2023). Data sourced from DWD Climate Data Center (2021). Figure S3: Strong positive correlation between PC1 values and (A) average temperature, (B) maximal temperature, (C) sum of dry days and (D) strong negative correlation between PC1 and mean precipitation (n = 631). Pearson correlation using ‘correlation’ from R package ‘correlation’ (Makowski et al. 2020). Data sourced from DWD Climate Data Center (2021). Figure S4: Trends in crop yield over time of moderately (green points) and strongly (blue triangles) pollinator‐dependent crops, as well as pollinator‐independent crops (orange squares) in two climatically different regions in Germany. Thin lines show the overall trend line for each pollinator dependency group compared to bold lines showing the respective crop type's trend. Solid lines represent significant trends as opposed to dashed lines for insignificant trends. Linear modelling using ‘lm’ from the R ‘stats’ package (R Core Team 2023). Data sourced from LfStat and LfStaD (2021) and R. Schätzl, J. Reisenweber and M. Sc [file ECE3-16-e73751-s001.docx]

# Supporting Information 1


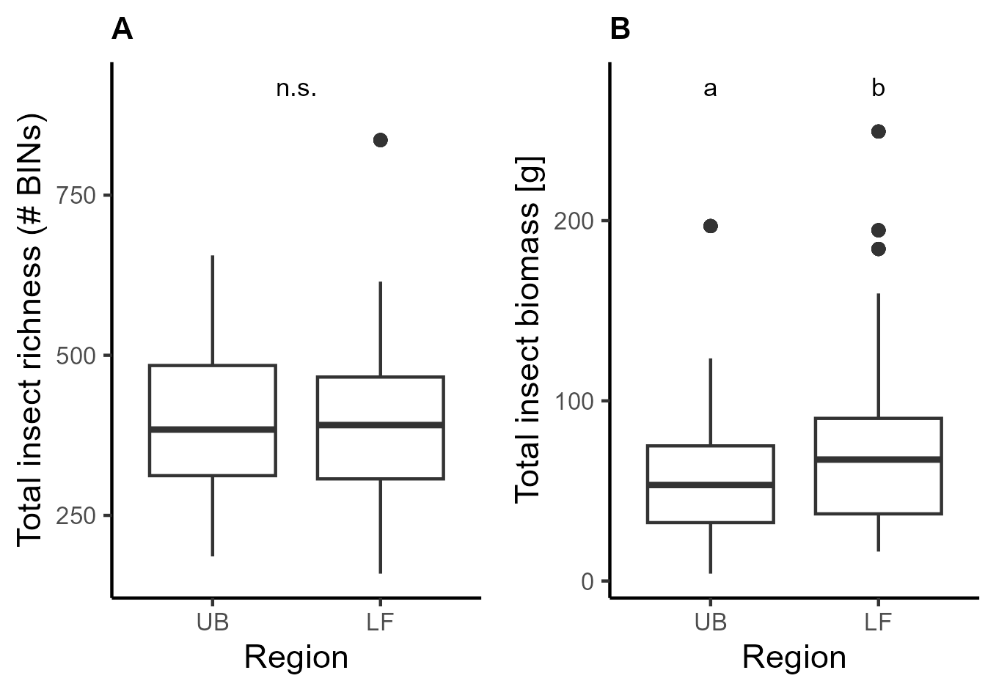

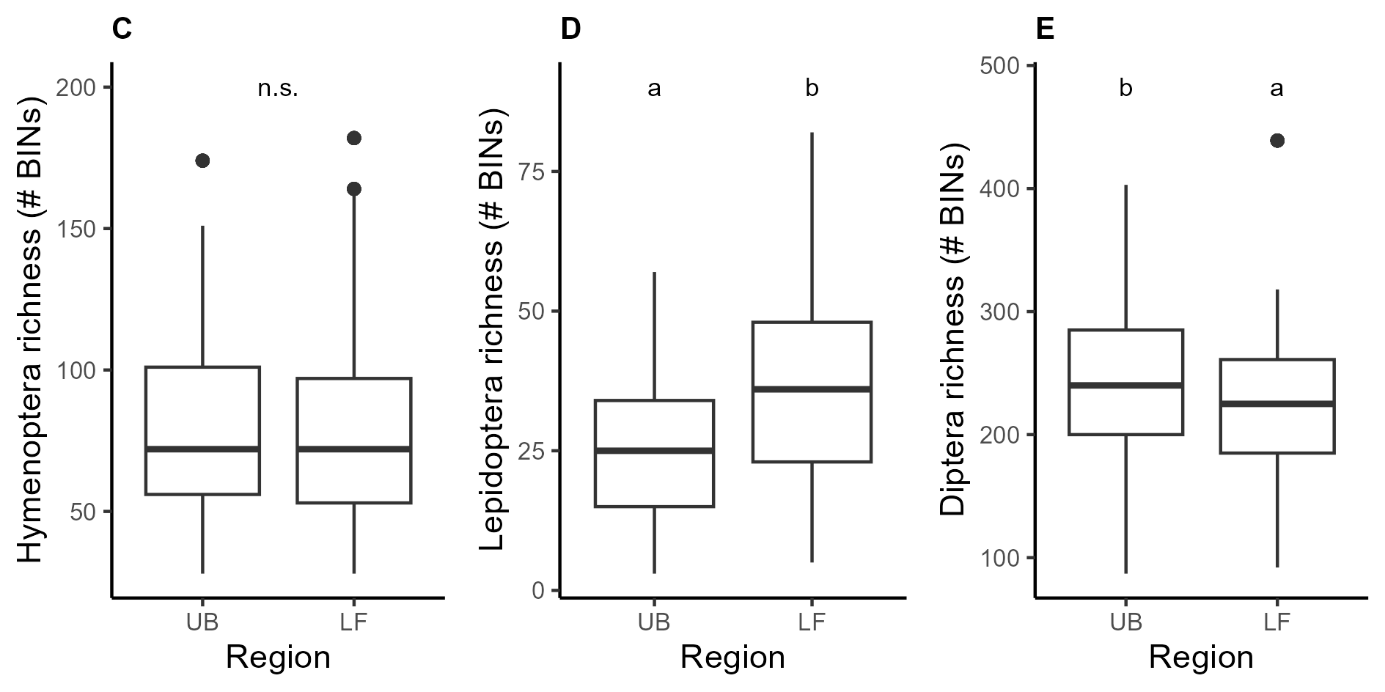


**Figure S1:** Malaise trap data collected by Uhler (2021) in 2019 shows (A) similar total insect richness and (B) Hymenoptera richness in the two study regions, i.e. cool-moist Upper Bavaria (UB) and warm-dry Lower Franconia (LF), while (C) total insect biomass and (D) Lepidoptera richness was higher in LF, and (E) Diptera richness was higher in UB (all p < 0.01; n = 696–968). Linear modelling using ‘lm’ and ‘anova’ from R package ‘stats’ (R Core Team, 2023).


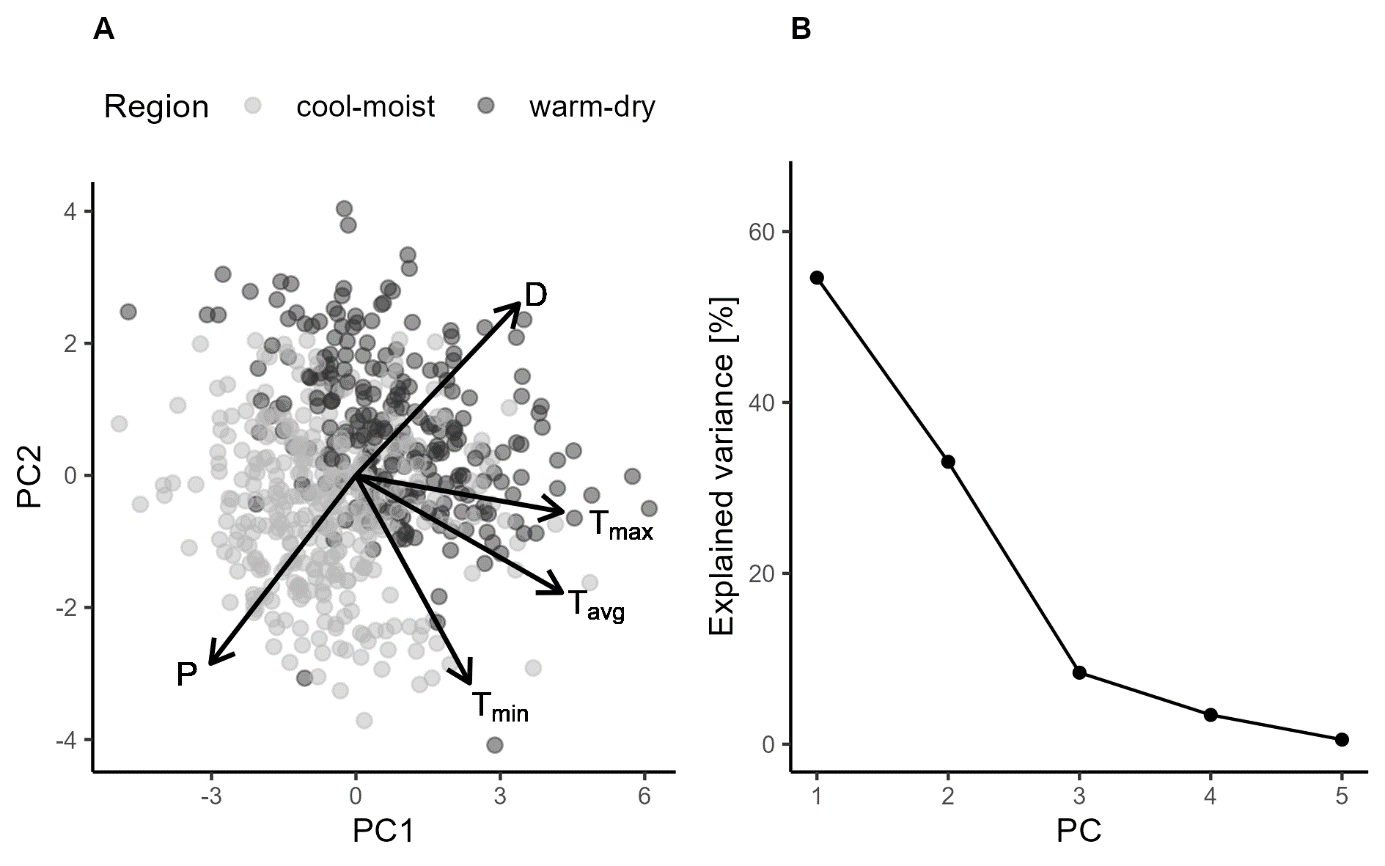


**Figure S2:** Clustering of climate records from the two study regions (cool-moist UB: light grey, warm-dry LF: dark grey) with the contribution of climate variables (D = sum of dry days; P = mean precipitation; T_avg_ = mean average temperature; T_max_ = mean maximum temperature; T_min_ = mean minimum temperature) based on their principal components 1 and 2 (A; n = 631) cumulatively explaining 88% of variance (B). Principal component analysis using ‘prcomp’ from R package ‘stats’ (R Core Team, 2023). Data sourced from DWD Climate Data Center (2021).


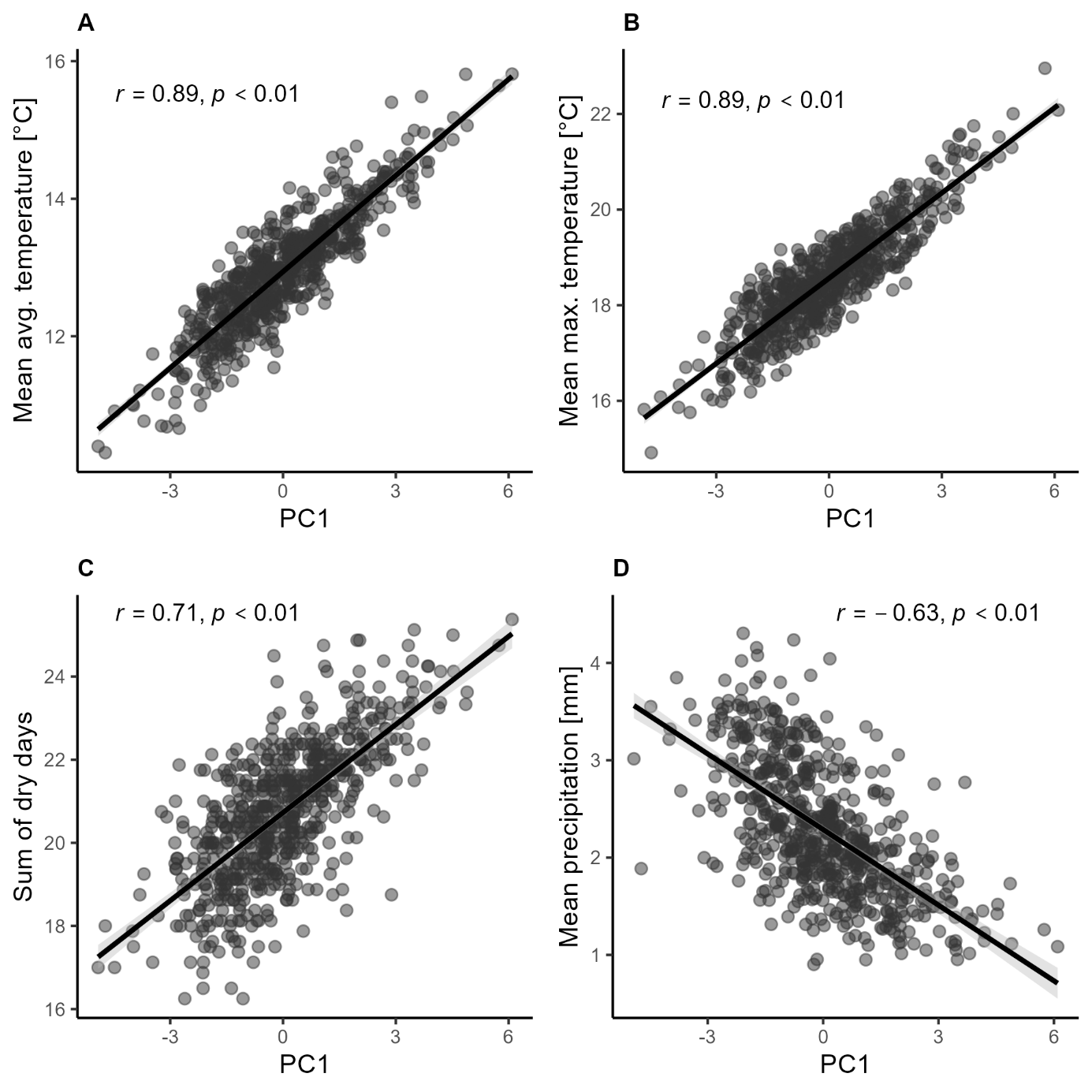


**Figure S3:** Strong positive correlation between PC1 values and (A) average temperature, (B) maximal temperature, (C) sum of dry days and (D) strong negative correlation between PC1 and mean precipitation (n = 631). Pearson correlation using ‘correlation’ from R package ‘correlation’ (Makowski et al., 2020). Data sourced from DWD Climate Data Center (2021).

**Supporting Information 2**

**
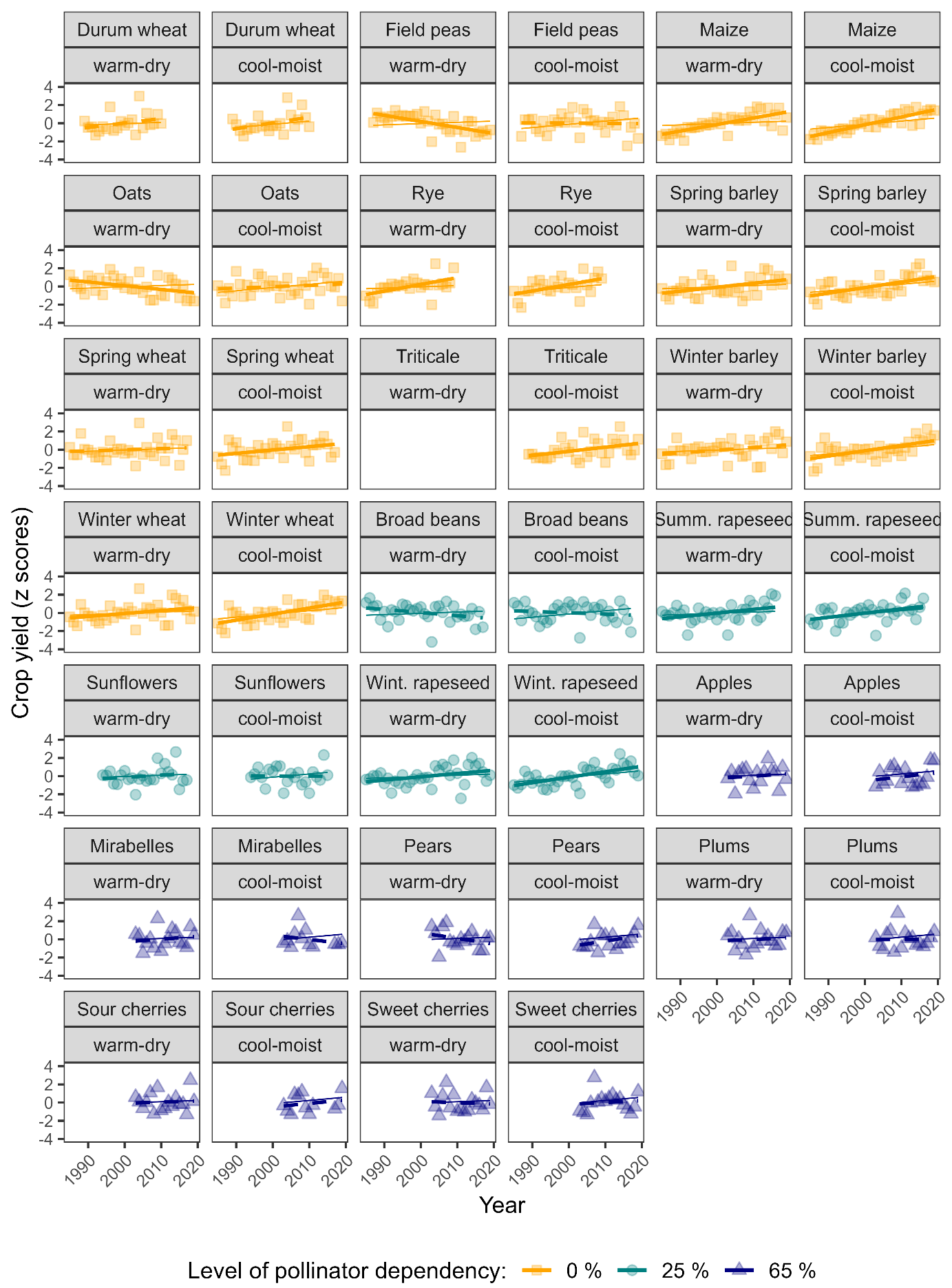
**

**Figure S4:** Trends in crop yield over time of moderately (green points) and strongly (blue triangles) pollinator-dependent crops, as well as pollinator-independent crops (orange squares) in two climatically different regions in Germany. Thin lines show the overall trend line for each pollinator dependency group compared to bold lines showing the respective crop type’s trend. Solid lines represent significant trends as opposed to dashed lines for insignificant trends. Linear modelling using ‘lm’ from the R ‘stats’ package (R Core Team, 2023). Data sourced from LfStat and LfStaD (2021) and R. Schätzl, J. Reisenweber and M. Schägger (pers. commun., 29 July 2021).


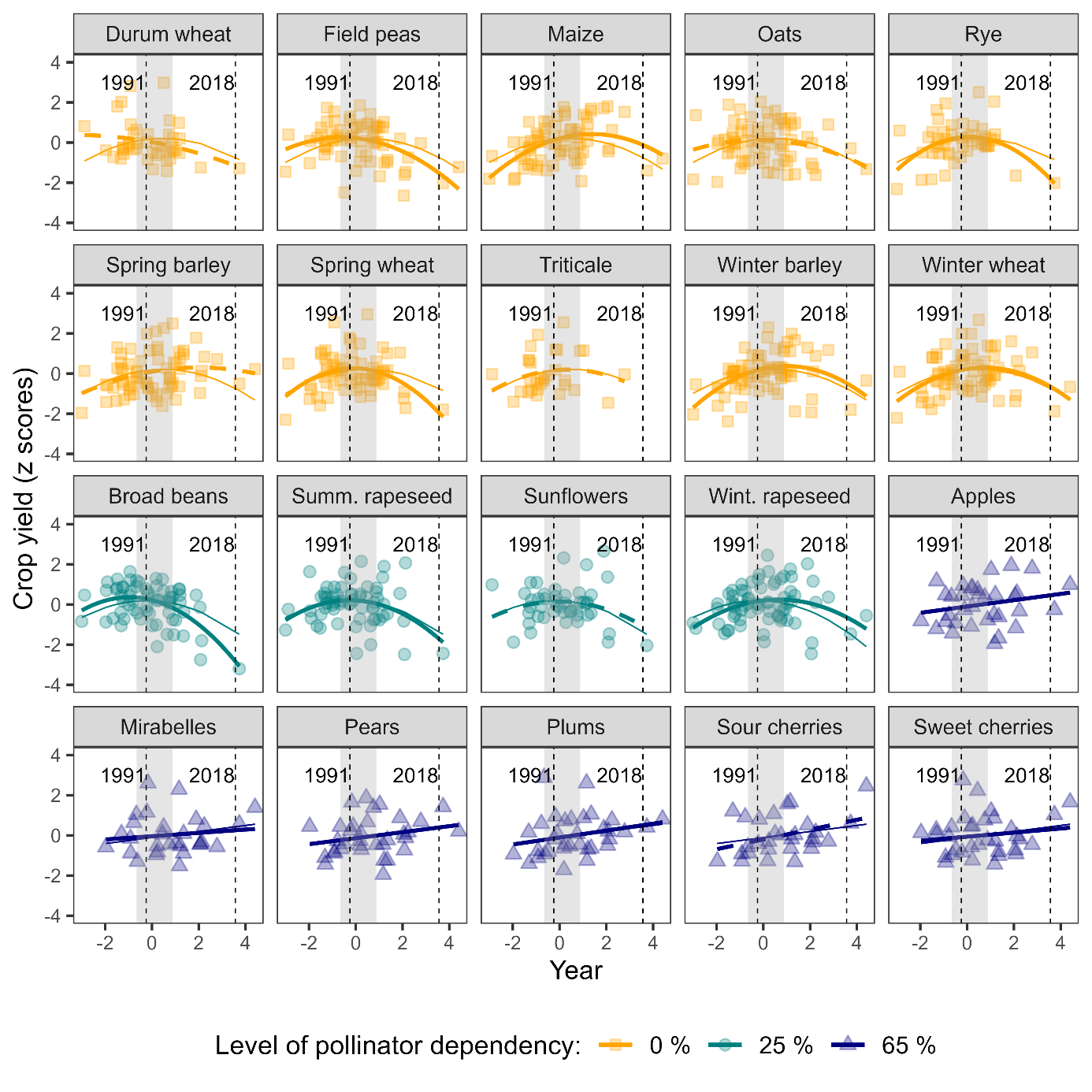


**Figure S5**: Relationship between climatic conditions and crop yields over time for moderately (green points) and strongly (blue triangles) pollinator-dependent crops as well as pollinator-independent crops (orange squares) across two climatically different regions in Germany. Dashed lines indicate regional PC1 average values for 1991 and 2018, two years with below and above long-term average temperature records on national scale, respectively (DWD Climate Data Center, 2021), while grey areas indicate regional PC1 average values across the dataset (R Core Team, 2023). Thin lines show the overall trend line for each pollinator dependency group compared to bold lines showing the respective crop type’s trend. Solid lines represent significant trends as opposed to dashed lines for insignificant trends. Linear modelling using ‘lm’ from the R ‘stats’ package (R Core Team, 2023). Yield data sourced from LfStat and LfStaD (2021) and R. Schätzl, J. Reisenweber and M. Schägger (pers. commun., 29 July 2021). Climate data underlying PC1 values sourced from DWD Climate Data Center (2021).

**References**

Bavarian State Statistical Office [LfStat], & Bavarian State Office for Statistics and Data Processing [LfStaD]. (2021). *Obsternte in Bayern: Ergebnisse der Ernte- und Betriebsberichterstattung über Baumobst [jährlich] [Fruit harvest in Bavaria: Results of the harvest and farm reporting on tree fruit [annually]]*. https://www.statistischebibliothek.de/mir/receive/BYSerie_mods_00000533

German Meteorological Service [DWD] Climate Data Center. (2021). *Daily station observations (temperature, pressure, precipitation, sunshine duration, etc.) for Germany* Version v21.3). https://opendata.dwd.de/climate_environment/CDC/observations_germany/climate/daily/kl/historical/

Makowski, D., Ben-Shachar, M., Patil, I., & Lüdecke, D. (2020). Methods and algorithms for correlation analysis in R. *Journal of Open Source Software*, *5*(51), 2306. https://doi.org/10.21105/joss.02306

R Core Team. (2023). *R: A language and environment for statistical computing*. In R Foundation for Statistical Computing. https://www.R-project.org/

Schätzl, R., Reisenweber, J., & Schägger, M. Hektarerträge Bayerns 1958–2019. Land - Regierungsbezirke - Landkreise [Hectare Yields of Bavaria 1958–2019. State - Administrative Districts - Counties]. In P. Prucker (Ed.).

Uhler, J. (2021). *Relationship of insect biomass and richness with land use along a climate gradient* Dryad. https://doi.org/10.5061/dryad.zkh1893bb
